# Atypical case of an orf virus infection in sheep

**DOI:** 10.1186/s12917-026-05750-y

**Published:** 2026-07-23

**Authors:** Anna Segna, Isaac David Portobanco Villalobos, Martin Ganter, Martin Ludlow, Wolfgang Baumgärtner, Katharina Manuela Gregor

**Affiliations:** 1 Department of Pathology, University of Veterinary Medicine, Hannover, Germany; 2 Clinik for Swine and Small Ruminants, University of Veterinary Medicine, Hannover, Germany; 3 Rearch Center for Emerging Infections and Zoonoses (RIZ), University of Veterinary Medicine, Hannover, Germany

**Keywords:** Contagious ecthyma, Dermatitis, Electron microscopy, Histology, Orf virus, Parapoxvirus ovis, Phylogenetic analysis, Podal form, Sheep, Tumor-like

## Abstract

**Background:**

Atypical manifestations with a podal distribution have been occasionally reported in the USA, the UK and Israel in the early 2000s. The lesions were characterised by a tumor-like progression with a lack of remission over months and seemed to affect mostly older lambs and adult sheep, and ultimately led to euthanasia. Here, we report a case from Germany with similar findings, raising the question of whether this presentation may represent a distinct syndrome associated with orf virus infection in adult animals.

**Case presentation:**

In February 2024, an eleven-month-old lamb showed extensive non-resolving proliferative lesions on all four legs around the coronary bands. After euthanasia, pathomorphological and molecular analyses of the affected area were performed. Histologically, the haired skin showed prominent vacuolation and swelling of keratinocytes of the stratum spinosum, reticular degeneration, and extensive epidermal proliferation with eosinophilic cytoplasmic inclusion bodies indicating a poxvirus infection. Transmission electron microscopy and PCR for the B2L and RPA gene confirmed an infection with the orf virus. Phylogenetic analysis revealed that the identified German orf strain was most closely related to an Iranian strain. The strains only differed by one amino acid (A98E) in the B2L open reading frame.

**Conclusions:**

The observed podal tumor-like lesion did not spontaneously resolve and ultimately led to euthanasia of the lamb due to severe lameness. Host and viral factors may play a role in this unusual presentation. The close genetic relationship between the German strain and the Iranian IRFH9 strain is noteworthy but remains elusive. Full- genome sequencing will be required to better understand the evolutionary origins of the viral strain. This appears to be the first report of an atypical manifestation of CE in Germany.

**Supplementary Information:**

The online version contains supplementary material available at 10.1186/s12917-026-05750-y.

## Background

Contagious ecthyma (CE), also known as contagious pustular dermatitis, sore mouth, scabby mouth and orf, is a zoonotic, localised infection and observed predominantly in young sheep and goats [1–3]. It is caused by the orf virus (ORFV), genus *Parapoxvirus*, family *Poxviridae*, subfamily *Chordopoxvirinae* [1–3]. While CE is self-limiting within 4–6 weeks and does not require specific treatment, it still results in notable economic challenges for livestock farmers due to its impact on productivity and its secondary complications [1, 4–6]. The impact is reflected in a reduced weight gain in suckling lambs, an increased risk of mastitis in ewes and a reduced quality of animal skin and wool [1, 5]. ORFV is usually transmitted by direct contact with infected animals or indirect contact with contaminated vehicles in the environment [7–10]. Mortality is usually a consequence of a secondary bacterial or parasitic infection [9–11]. The high environmental stability of the virus is primarily due to the protection within dried scabs or crusts, which serve as reservoirs of infectious virus particles and enable long-term persistence of the disease within the flock. Currently, CE is primarily controlled through vaccination or hygiene measures, although with variable levels of success [12].

In addition to sheep and goats, CE has been reported in camelids, other ruminant species (e.g., serows), and domesticated companion animals such as cats and dogs [13–16]. Also, zoonotic infections in humans have been reported in individuals (farmers and veterinarians) who came into direct or indirect contact with skin lesions of the infected animals [13]. Typically, gross lesions occur on the lips and may also involve the nostrils, eyelids, and ears, depending on the site of the viral inoculation [9, 13]. Infected lambs may transfer the infection to the teat and the udder of the ewe [6, 9]. In addition, lesions also occur on the coronary band, the perineum, the vulva, and, less commonly, the tail dock site or the head [9, 10]. Ulcerative CE-lesions are occasionally observed on the gums, the tongue and the palate, which can have regulatory relevance, particularly in foot-and-mouth- disease-free countries with eradication programs [9]. On rare occasions, the extent of the lesions to the inner organs, including the oesophagus and the rumen, has been described [9]. Secondary bacterial infections can lead to secondary systemic signs such as fever, apathy, or swollen regional lymph nodes [9, 10, 17].

In the initial stages, CE is characterised by erythematous macules that evolve into vesicles [9]. These vesicles progress into pustules, which eventually dry out to form thick brown-grey crusts [9]. Histologic hallmarks are vacuolation and swelling of keratinocytes in the stratum spinosum, reticular degeneration, an extensive epidermal proliferation and the presence of intraepidermal microabscesses [9]. A thick crust, characterised by a marked para- and orthokeratotic hyperkeratosis, is also commonly observed [9]. CE is recognised as a localised, self-limiting disease with a low mortality rate and one or multiple lesion sites in areas that are prone to trauma and virus entry (e.g., lips, ears, and distal extremities) [7, 9, 10, 13]. The cause for higher mortality rates in young lambs and kids is mostly due to secondary bacterial infection [7, 9, 10, 13].

This case report describes an eleven-month-old lamb with a severe, painful, proliferative dermatitis confined to all four legs, which persisted for several months without resolution, ultimately leading to euthanasia. A comparison to previous case reports may suggest a CE-related syndrome in adult sheep and older lambs. This case report aims to document a clinically relevant case, contribute to the existing body of evidence, and raise awareness of this clinical presentation. This appears to be the first report in Germany describing an atypical manifestation with podal distribution of CE in sheep.

## Case presentation

**Fig. 1:**
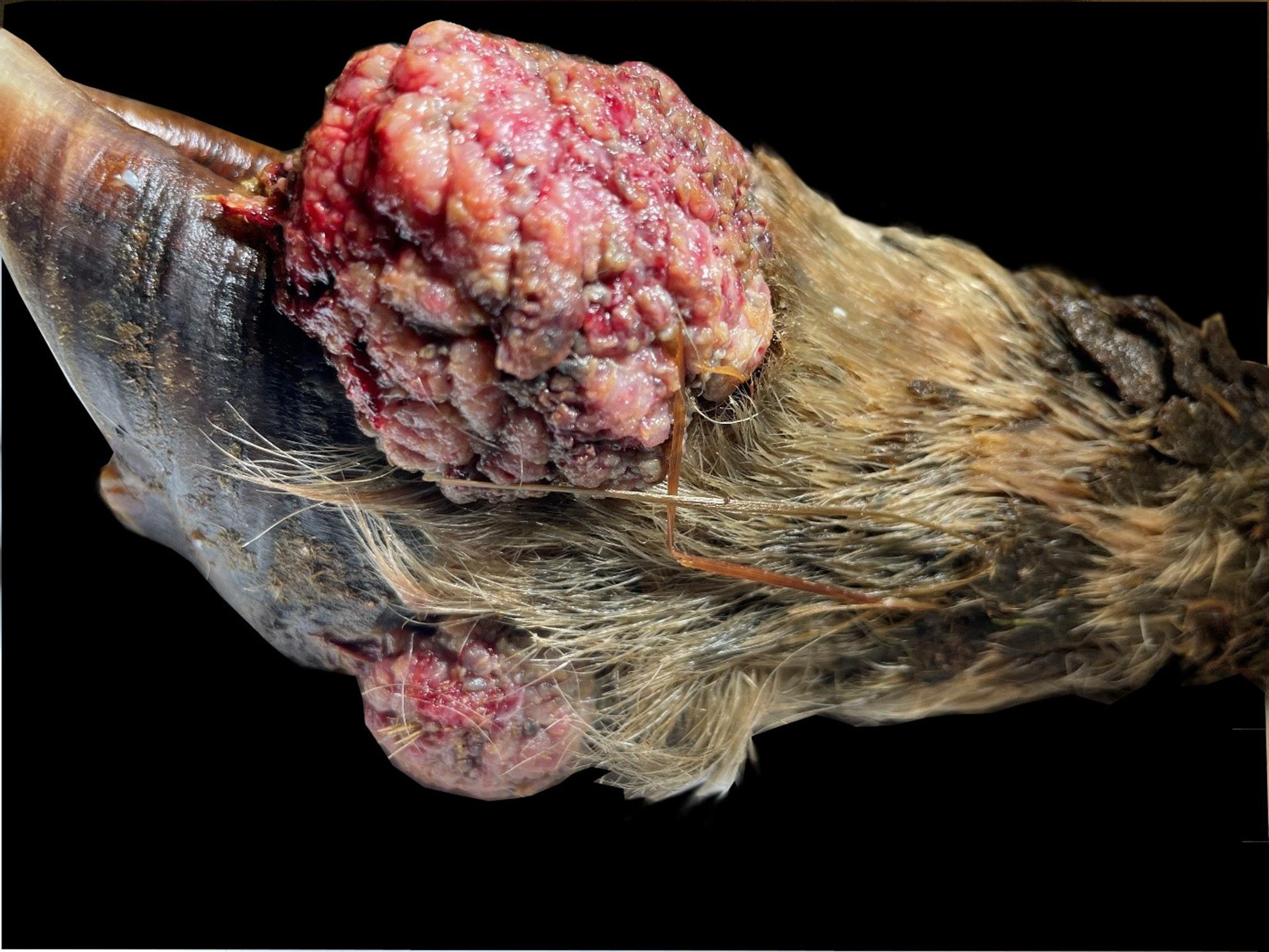
Right front leg of the lamb affected with CE. Proliferations are seen on the dorsal and on the flexion part of the leg at the coronary band

In February 2024, an eleven-month-old lamb with severe proliferative skin lesions confined to the legs was presented by the shepherd during a routine farm visit. The lamb was emaciated, showed severe lameness, and was mostly resting in sternal recumbency. The proliferations on the claws showed no signs of improvement or remission for months. Similar lesions were observed in its sibling. Due to animal welfare reasons, the lamb was stunned with a bolt gun and subsequently bled by opening the jugular veins. The podal lesions were submitted for further investigation. However, the sibling was not investigated further. The flock consists of about 843 ewes, 41 sires, 2226 gimmers and 1526 lambs. The sheep are primarily crossbred, consisting of Texel and Black-Headed Mouton ewes mated predominantly with Swifter sires. CE is endemic in this flock. Vaccination is practised with the attenuated live vaccine Ecthybel^®^ (Boehringer Ingelheim, formerly Merial, Laboratoire Porte des Alpes, Saint-Priest, France) in motherless reared lambs after a significant outbreak of CE in motherless reared lambs in early 2002 and 2003, which was linked to the spread via plastic teats. However, the investigated lamb was reared with its mother and was unvaccinated. Since 2004, sporadic cases of tumor-like manifestations of CE affecting the distal extremities have been observed in older lambs and adults (Fig. 1). In addition, some animals developed lesions on the head. Affected animals generally exhibited a good weight gain, and no other remarkable clinical findings were noted. In the neighbouring flock isolated cases of a tumor-like podal form of CE have also been reported.

**Fig. 2:**
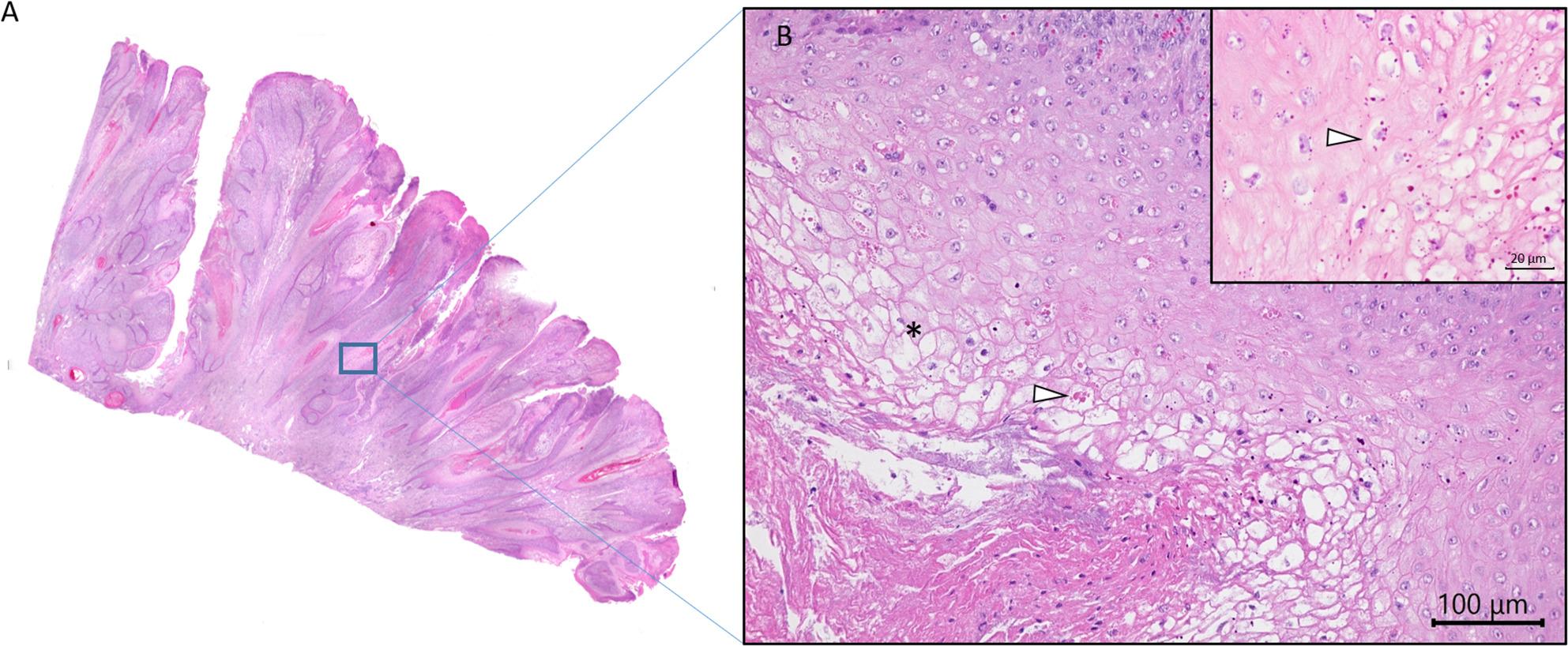
Histologic findings of a lamb with proliferative dermal lesions on the distal limbs. **A** The ulcerated proliferation is characterized by severe epidermal hyperplasia, parakeratotic hyperkeratosis and anastomosing rete ridges with a dermal fibrous core with marked histiocytic inflammation. **B** Keratinocytes with ballooning degeneration (asterisk) and multiple cytoplasmatic eosinophilic inclusions of about 2 µm in diameter (white arrowhead). Insert: Cytoplasmatic eosinophilic inclusion within keratinocytes. Hematoxylin and eosin stain

After submission, a sample of the podal lesion (Fig. 1) was fixed in 10%-buffered formalin for 24 h and embedded in paraffin wax (FFPE). Thereafter, specimens were cut into 2 μm thick sections and stained with hematoxylin and eosin (H&E) for histopathological examination. Histologically, the largely ulcerated haired skin showed a focal thickening of the epidermis characterised by severe hyperplasia, parakeratotic hyperkeratosis with acanthosis and anastomosing rete ridges, which was supported by a broad core of dermal fibrous connective tissue. A serocellular crust with numerous viable and degenerated neutrophilic granulocytes, as well as the formation of microabscesses, fibrin, and coccoid bacterial colonies, was evident in the superficial epidermis. Within the stratum spinosum, multiple keratinocytes were markedly swollen, showed loss of eosinophilia and were characterised by ballooning degeneration. Dissociated cells with a hypereosinophilic cytoplasm and pyknotic nuclei, indicating necrosis, were occasionally found. In addition, cytoplasmic small eosinophilic inclusion bodies were frequently noticed (Fig. 2). The fibrous core of the podal lesion showed a marked infiltration by macrophages, lymphocytes and a few plasma cells as well as neovascularisation with multifocal mild to moderate hemorrhages and a mild diffuse edema.

**Fig. 3:**
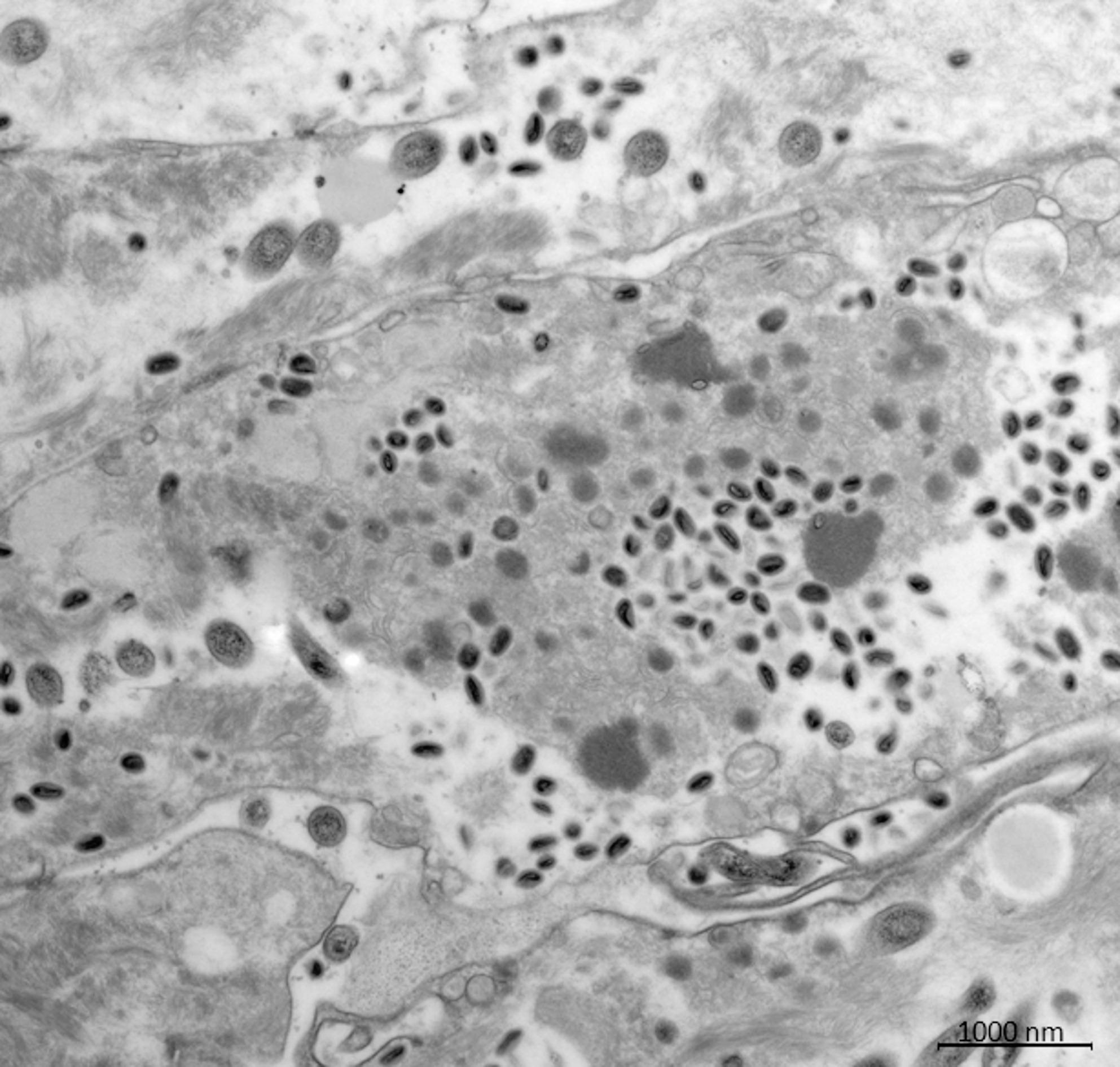
Transmission electron microscopy of a lamb with proliferative dermal lesions on the distal forelimbs. Keratinocytes of the haired skin contain numerous cytoplasmic viral particles, viral factories and A-type inclusions. Uranyl acetate, bar = 1000 nm

For transmission electron microscopy (TEM), FFPE specimens were cut into pieces of approximately 1 mm thickness. Samples were washed in 0.1 M cacodylate buffer with intermediate fixation in 5% glutaraldehyde overnight and incubation in 1% osmium tetroxide for 2 h. Skin specimens were then dehydrated in an ascending alcohol series, immersed in propylene oxide for 30–60 min and incubated overnight in propylene oxide with epon resin before incubation in pure epon for 30–60 min. Thereafter, samples were embedded in epon with 2,4,6-tridimethylaminomethyl phenol (DMP-30) and incubated in an oven for six days at increasing temperatures from 35 °C to 65 °C. Ultrathin sections of 82 nm were stained positive with 0.3% uranyl acetate for 20 min in the dark and lead nitrate (II) for 10 min. TEM showed evidence of numerous ovoid-shaped, encapsulated inclusion bodies of approximately 220–300 nm length and 140–170 nm width in the cytoplasm of epithelial cells. In addition, several epithelial cells contained viral factories and A-type inclusion bodies (Fig. 3).

An ORFV-specific PCR was performed to confirm an ORFV infection using previously described primers (Parapox_Orf1, 5’-CGCAGACGTGGCTGAGTACGT-3’; Parapox_Orf2, 5’-TGAGCTGGTTGGCGCTGTCCT-3’) targeting the conserved ORFV-RPA gene resulting in a 140 bp amplicon [18]. DNA was extracted from frozen tissue using a DNeasy Blood & Tissue Kit (Qiagen) according to the manufacturer’s instructions. PCR amplification of the ORFV-specific sequence was performed with Q5 High-fidelity DNA polymerase kit (New England Biolabs, NEB) with an annealing temperature of 68 °C. The full-length open reading frame (1137 bp) of the B2L gene was then also amplified using Q5 polymerase using previously reported primers (OVB2LF1, 5’-TCCCTGAAGCCCTATTATTTTTGT 3’; OVB2LR1, 5’ GCTTGCGGGCGTTCGGACCTTC-3’) (PMID: 16777357) at an annealing temperature of 61 °C. Both amplicons were purified using the Monarch Gel Extraction kit (NEB) and Sanger sequenced (Microsynth Seqlab) using the forward and reverse primers used in the respective PCR reaction. Amino acid sequences of complete B2L open reading frames were aligned using the T-coffee multiple sequence alignment program [19] and visualized using ESPript 3.0 [20].

**Fig. 4:**
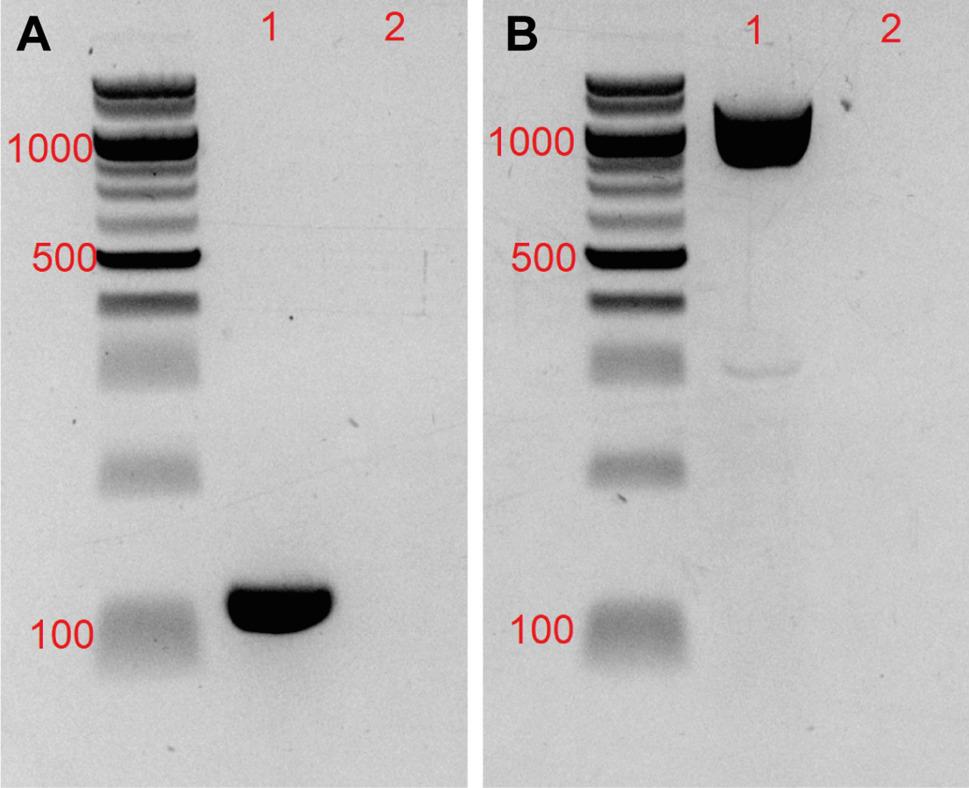
PCR result for Orf virus. **A** Amplification of a 140 bp amplicon of the ORF-RPA gene (lane 1) from DNA extracted from the proliferative dermal lesion of a lamb in Germany. No specific band was visible in a PCR reaction without template DNA (lane 2). **B** Amplification of a 1137 bp amplicon of the B2L gene (lane 1). No specific band was visible in a PCR reaction without template DNA (lane 2). Sizes of bands were determined be reference to a 100 bp DNA ladder (NEB)

**Fig. 5:**
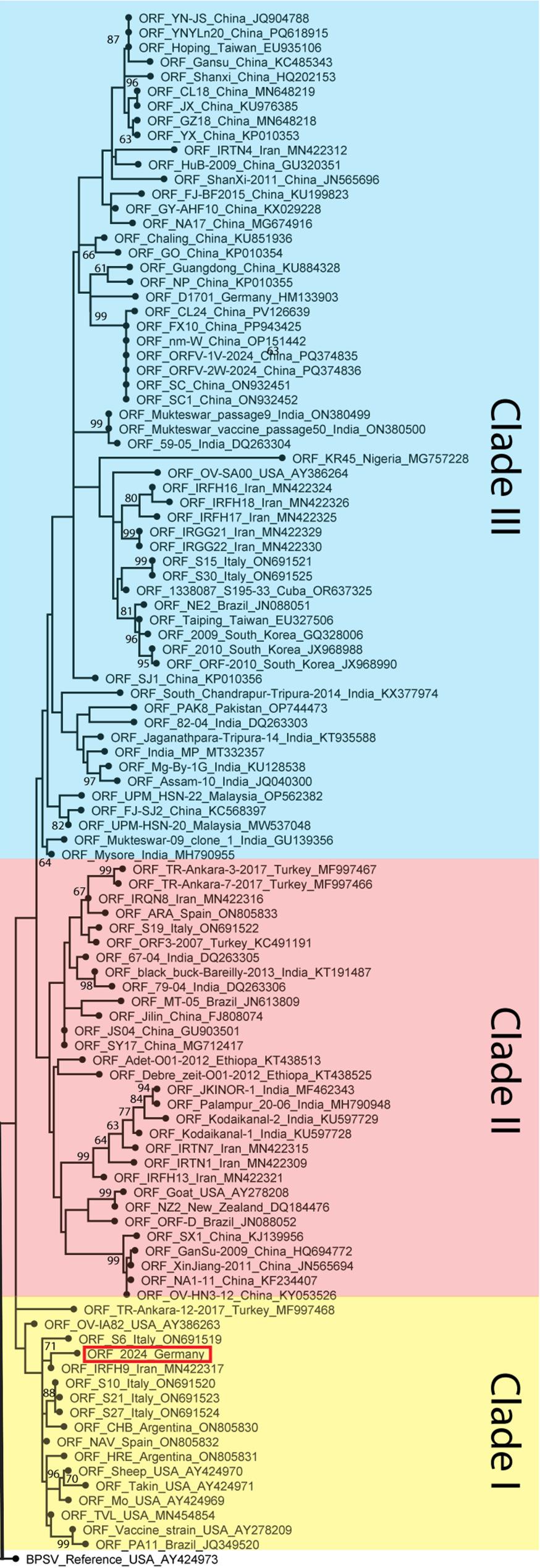
Maximum likelihood tree based on 105 complete ORF B2L open reading frame sequences (1137 bp). Phylogenetic analysis was performed using MEGA X using the Tamura 3-parameter model with gamma distribution and invariable sites (T92+G+I) with a total of 1000 bootstraps. Three separate clades are highlighted in yellow (clade I), pink (clade II) and blue (clade III). The German ORF sequence analysed in this study is indicated with a red box. Tip labels indicate the strain designation, country of origin and the GenBank accession number. A reference strain of Bovine papular stomatitis virus (BPSV; GenBank accession no. AY424973) was used as an outgroup. The scale bar indicates the number of substitutions per sites with branch lengths being proportional to genetic distances. Bootstrap values ≥60% are shown adjacent to the major nodes

The evolutionary relationship between ORFV strains was investigated using the complete sequences of the B2L open reading frame. A multiple sequence alignment was generated with the sequences downloaded from GenBank using the MAFFT version 7. The Tamura-3-parameter model with a discrete Gamma distribution (+ G) and some evolutionarily invariable sites (+ I) was calculated as the best fit with MEGA X for all alignments and used for the Maximum Likelihood method with 1000 bootstraps. MEGA X was used to perform the analysis with a total of 106 sequences. Branch lengths illustrate the number of nucleotide substitutions per site as indicated by the scale bar. The presence of ORFV in the proliferative dermal lesion was confirmed by performing an ORFV-specific PCR targeting a highly conserved region of the ORF-RPA gene, which encodes for RPO132, a major component of the viral RNA polymerase. This resulted in a 140 bp amplicon (Fig. 4A), Sanger sequencing of which showed 100% nucleotide similarity to multiple ORFV strains upon analysis using NCBI BLAST (Basic Local Alignment Search Tool). A second PCR was performed to amplify the 1137 bp open reading frame of the B2L gene, which encodes for a major envelope protein to facilitate phylogenetic analysis (Fig. 4B). Sanger sequencing of the amplicon showed 98.35 to 99.20% homology to multiple ORFV strains upon BLAST analysis. The evolutionary relationship of the German ORFV strain was delineated by performing a phylogenetic analysis with the complete B2L sequences available on GenBank (Fig. 5). This showed that the German ORFV strain grouped with strains of diverse geographical origins (clade I) including the older OV-IA82 strain from the USA (GenBank accession no. AY386263) and claded separately from well characterized ORFV lineages which have previously been reported which include NZ2 from New Zealand (GenBank accession no. DQ184476) (clade II) and OV-SA00 from the USA (GenBank accession no. AY386264) (clade III) (Fig. 5). The German ORFV strain was found to be most closely related to an ORFV strain (IRFH9, GenBank accession no. MN422317), which was derived from a mouth scab collected from a sheep in Iran in 2019. Comparison of B2L amino acid sequences from Clade I strains confirmed that the German ORFV strain was most closely related to this Iranian strain, with only one amino difference (A98E) between the two strains (Figure S1).

## Discussion and conclusions

This case report describes an atypical manifestation of CE in a sheep in Germany. In the presented case, lesions were predominantly confined to the legs and showed no signs of remission. While the lack of a spontaneous remission is described as an atypical manifestation of CE, the podal distribution, which is sometimes accompanied by head lesions, also appears to be a consistent pattern reported in atypical cases from the USA, the UK, and Israel [21–23]. The Israeli report from 2000 noted that most animals showed lesions confined to the legs, with only twelve ewes of approximately seventy affected animals also exhibiting forehead lesions [22]. Similarly, the report from the USA in 2001 documented five sheep with painful, extensive and proliferative, wart-like lesions confined to the legs, with two of five animals also showing head lesions [21]. In Boer and Boer-crossed goats, persistent ORFV infections are also described, which are characterised by a severe proliferative dermatitis [17]. However, lesions were distributed not only to the legs but also to the ventral aspects of the thoracic, abdominal and inguinal region [17]. Nevertheless, it has been reported that lesions in the interdigital space and above the coronet were present in most kids [17]. The expansive distribution was suggested to be linked to trauma due to the exposure of the affected body sites to the rough vegetation in Texas [17]. The clinical presentation in Boer goats and Boer-crossed goats is discussed to be the result of individual susceptibility factors within some animals [17, 24].

Macroscopically and histologically, the lesions in this case were mostly consistent with the classical presentation of CE in small ruminants, progressing from macules to vesicles and pustules, culminating in thick brown-grey crusts, yet with a more severe manifestation and a cauliflower-like appearance [9]. This appears to be consistent with the previously reported cases [21, 22]. The formation of the crust seen in this case corresponds to an advanced stage of the disease. No gross lesions were evident in other common anatomical sites, and the prominent tumor-like podal proliferation was especially remarkable [9, 13, 25]. The podal lesions presented the histologic hallmark features, such as a marked epidermal hyperplasia, a ballooning of keratinocytes, and the presence of eosinophilic, cytoplasmic inclusions [9]. The marked inflammation and ulceration were most likely due to secondary bacterial infection. Similar findings have been observed in previous atypical cases.

The presented lamb matched the age pattern of previous cases, where affected sheep ranged from twelve months to two years, with only one lamb aged four months [21]. Boer and Boer-crossed goats affected by a persistent ORFV infection were aged two to six months. The overall significance of this observation for the pattern observed in sheep remains inconclusive and warrants further investigation [17]. It has been discussed whether adult sheep may experience a higher risk of trauma to the legs due to environmental exposure. This may facilitate viral entry as discussed in the Boar goat case report, which may contribute to the observed podal localisation in the presented and the historical cases. However, this remains speculative and needs to be addressed in future studies.

The presented lamb was unvaccinated. Nevertheless, vaccination is practised in the flock. In the report from the USA in 2001, the vaccination status was not mentioned, whereas the Israeli report from 2000 noted that none of the animals had been vaccinated [21, 22]. In contrast, all affected Boer goats were vaccinated at either one day or two weeks of age with a live vaccine strain [17]. The authors noted that experimental inoculation of an isolate from severe cases into naïve young goats resulted in only mild, transient lesions [26]. Although the possibility of a vaccine origin was considered, results indicated substantial differences between the vaccine strain and the ORFV strain, and thus this hypothesis was not substantiated [26]. Furthermore, it has been suggested that host factors might influence the severity and persistence of the disease in susceptible individuals, and that the virus strain may also play a role in determining susceptibility among different species [17, 27]. This finding aligns with a study that characterised ORFV isolates from a musk ox, a Sichuan takin, and domestic Shetland sheep in a zoo [27].

Immunosuppressive co-morbidities may contribute to the atypical manifestation, as described for the experimental infection with *Anaplasma phagocytophilum*, thermal injuries or co-infection with papilloma virus [21]. Nonetheless, no evidence of papillomavirus was found in previous reported cases [21]. The bloodwork was unremarkable, and secondary infection of the lesions was reported infrequently. In this case, the presence of Papillomavirus was not observed via TEM. However, the lamb was not specifically tested for other co-morbidities. Thus, the existence and the contribution of immunosuppressive co-morbidities to the observed manifestation cannot be ruled out.

The clinical progression in the lamb was characterised by a lack of spontaneous remission over months, which is atypical for classical CE, as it typically resolves spontaneously [21]. The course of CE shows similarities to the cases from the USA, where lesions were more severe compared to classical CE lesions and also did not resolve spontaneously [21]. In that report, animals were euthanised due to severe lameness without clinical improvement after local and systemic treatment with antibiotics and non-steroidal inflammatory drugs [21]. The Israeli report did not discuss lesion persistence or clinical progression [21, 22], but the severe proliferative and verrucous lesions, mostly confined to the legs, were observed across all four flocks [22]. In the reports concerning Boer goats, lesions in kids affected by persistent infection resulted in euthanasia [17, 25]. Lymphadenomegalie in all kids and a suppurative arthritis and premature thymic involution in some kids were additionally reported. As in the presented case, no other abnormal physical examination findings were reported in the cases from the USA or Israel [21, 22]. A consistent feature appears to be the poor clinical outcome associated with the atypical podal manifestation. Thus, severe lameness is a prominent factor contributing to the decision to euthanise affected animals.

The phylogenetic analysis based on complete B2L sequences revealed that the German ORF virus strain is closely related to strains from geographically diverse regions, including the USA and Iran, which share a common origin. The placement of the German strain in this clade, alongside both older and more recent clinical isolates, indicates an ongoing viral transmission between dispersed geographical regions. The close genetic relationship between the German strain and the Iranian IRFH9 strain is particularly noteworthy. However, full genome sequencing is required to better understand the evolutionary origins of this strain to determine if there are any unique features to the coding sequences, such as sequence deletions or evidence of recombination. B2L is an appropriate marker for confirmation and broad phylogenetic placement of ORFV isolates; however, its conserved nature limits its utility for fine-scale epidemiology, transmission inference, or evolutionary timing.

Tumor-like lesions associated with ORFV have also been reported in humans, commonly referred to as giant orf lesions, among other names [28]. Recent findings indicate that the viral vascular endothelial growth factor (vVEGF) in ORFV, which is a key pathological molecular factor encoded by the virus, interacts with host factors such as the vascular endothelial growth factor receptor 2 and may play a synergistic oncogenic role [29]. A study investigated pathogenic molecular factors encoded by ORFV and their link to the tumor-like growth in naturally infected young lambs and goats aged ten to twenty weeks [29]. However, predominantly oral lesions were described [29]. The animals were selected based on the severity and the tumor-like appearance of the lesions and were sourced from seven different farms [29]. However, the viral genome was not further investigated, leaving it unclear whether the lesions stem from the same viral strain [29]. Thus, vVEGF may also play a role in the atypical manifestation observed in the presented case, as in previous cases. Still, further detailed studies are needed to confirm these findings.

In conclusion, the observed podal tumor-like lesion did not spontaneously resolve and ultimately led to euthanasia of the lamb, as has been reported in previous cases. There are indications that the atypical manifestation with podal distribution may be the result of complex interactions between host and viral factors. However, the underlying cause of the podal distribution remains inconclusive and warrants further investigation. The number of reported cases is very limited for a final pathogenetic conclusion and interpretation. The close relationship to the Iranian strain is particularly noteworthy, but phylogenetic analysis allows no further interpretation with respect to the atypical manifestation with podal distribution. To elucidate genomic variation, tissue tropism, virulence, and clinical variability, detailed analyses, such as full genome sequencing and in vitro studies, are essential. The atypical manifestation with podal distribution may be considered an ORFV-related syndrome in older lambs and adult sheep, which might occur as a solitary lesion or in combination with other localised changes. Future studies should focus on atypical, non-resolving manifestations to improve the understanding of the underlying mechanisms and the viral dynamics of CE [21]. At last, occurrence of CE confined to the distal extremities should be considered as a differential diagnosis of persistent proliferative lesions in the podal area [21, 22, 30].

## Supplementary Information

Supplementary Material 1. Figure S1: Multiple alignment of the amino acid (aa) sequence of the major envelope glycoprotein (B2L) of ORF virus. A multiple sequence alignment of B2L aa sequences from clade I (all representatives), clade II (ORF_NZ2_New_Zealand_DQ184476) and clade III (ORF_OV_SA00_USA_AY386264); ORF_D1701_Germany_HM133903) was prepared using T-coffee (http://tcoffee.crg.cat./apps/tcoffee/do:regular). The output file from T-coffee was formatted using ESPript 3.0 (http://espript.ibcp.fr/ESPript/ESPript/index.php).

Supplementary Material 2. Figure S2: PCR result for Orf virus (Fig. 4 uncropped).

## Data Availability

The sequence data in the current study is available in the GenBank repository under the GenBank accession number PX920947. The datasets used and/or analysed during the current study are available from the corresponding author on reasonable request.
