# Supplementary figures and images for: Atypical case of an orf virus infection in sheep
Source: BMC Vet Res. 2026 Jul 23;22:441. doi: 10.1186/s12917-026-05750-y (PMC13401306; doi:10.1186/s12917-026-05750-y)

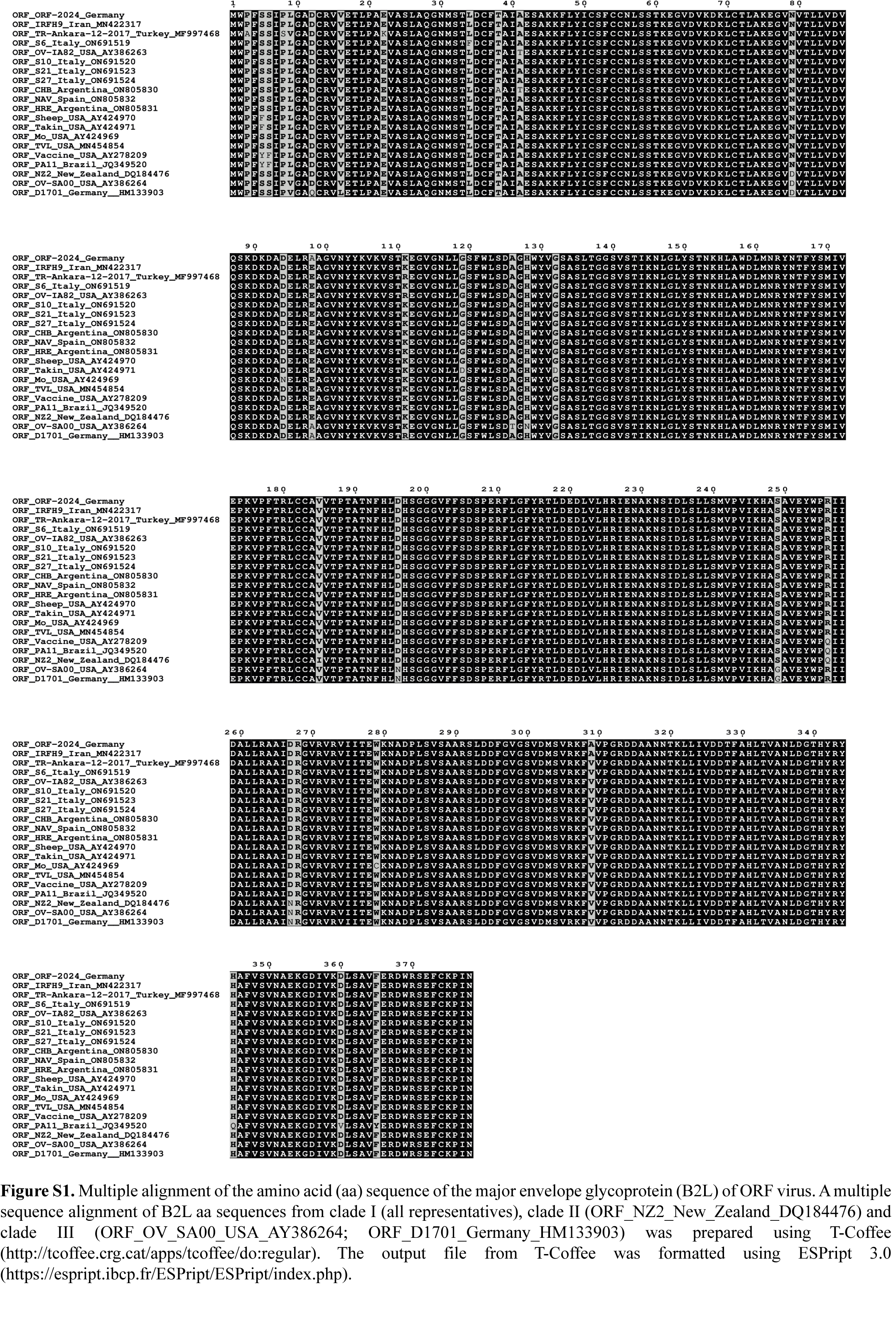

Supplement: Supplementary file 1 — Supplementary Material 1. Figure S1: Multiple alignment of the amino acid (aa) sequence of the major envelope glycoprotein (B2L) of ORF virus. A multiple sequence alignment of B2L aa sequences from clade I (all representatives), clade II (ORF_NZ2_New_Zealand_DQ184476) and clade III (ORF_OV_SA00_USA_AY386264); ORF_D1701_Germany_HM133903) was prepared using T-coffee (http://tcoffee.crg.cat./apps/tcoffee/do:regular). The output file from T-coffee was formatted using ESPript 3.0 (http://espript.ibcp.fr/ESPript/ESPript/index.php). [file 12917_2026_5750_MOESM1_ESM.tif]

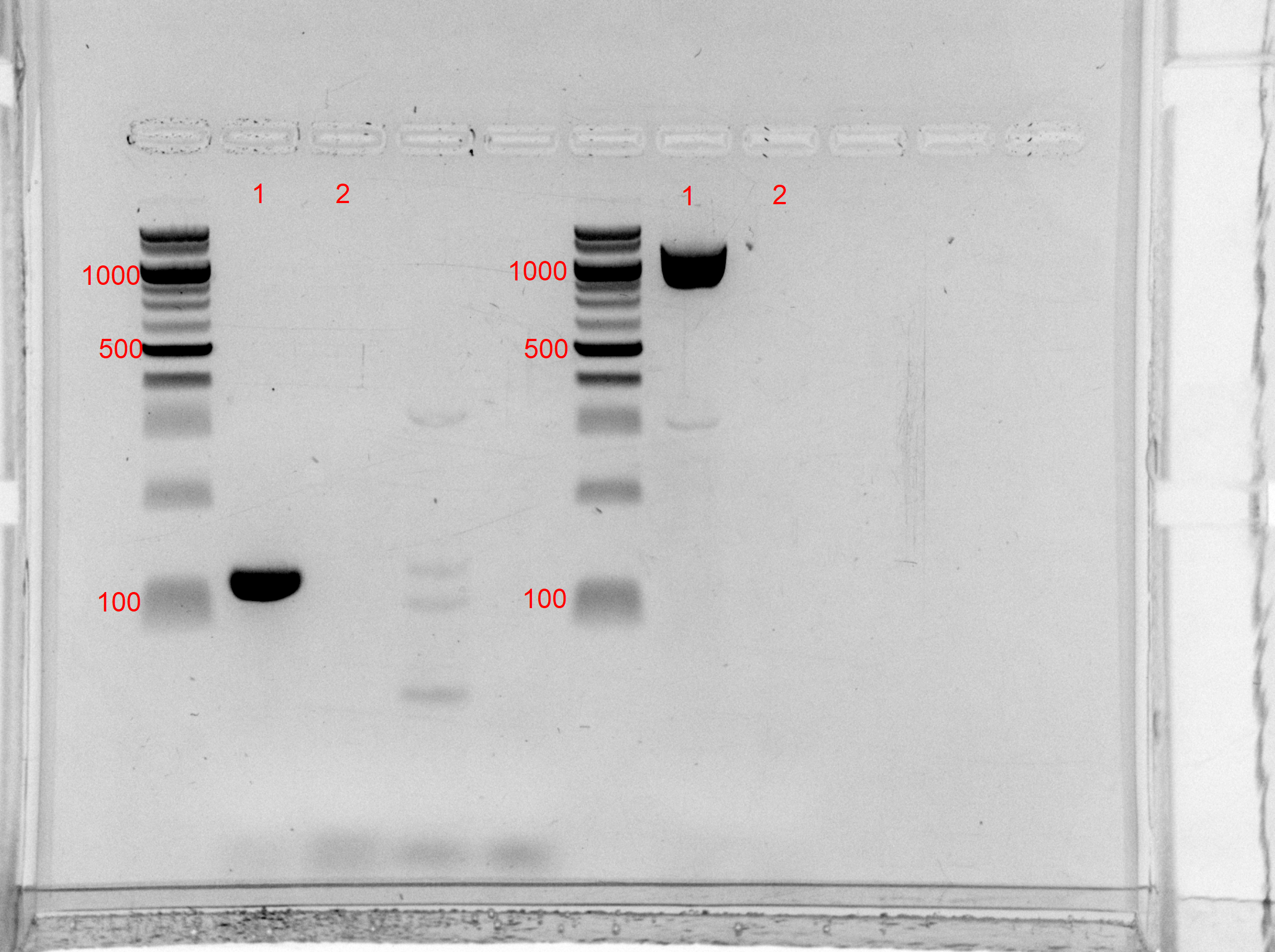

Supplement: Supplementary file 2 — Supplementary Material 2. Figure S2: PCR result for Orf virus (Fig. 4 uncropped). [file 12917_2026_5750_MOESM2_ESM.tif]
